# Effect of Eleutheroside E on an MPTP-Induced Parkinson’s Disease Cell Model and Its Mechanism

**DOI:** 10.3390/molecules28093820

**Published:** 2023-04-29

**Authors:** Yi Yao, Caiyu Liao, Honghao Qiu, Lishan Liang, Wenying Zheng, Liyan Wu, Fanxin Meng

**Affiliations:** School of Pharmacy and Food Science, Zhuhai College of Science and Technology, Zhuhai 519041, China

**Keywords:** eleutheroside E, Parkinson’s disease, 1-methyl-4-phenyl-1,2,3,6-tetrahydropyridine, apoptosis

## Abstract

This research investigated the effects of eleutheroside E (EE) on the 1-methyl-4-phenyl-1,2,3,6-tetrahydropyridine (MPTP)-induced Parkinson’s disease cell model and its mechanism. Methods: To create a cell model of Parkinson’s disease, MPTP (2500 μmol/L) was administered to rat adrenal pheochromocytoma cells (PC-12) to produce an MPTP group. Selegiline (50 μmol/L) and MPTP had been administered to the positive group beforehand. The eleutheroside E group was divided into low-, medium-, and high-concentration groups, in which the cells were pretreated with eleutheroside E at concentrations of 100 μmol/L, 300 μmol/L, and 500 μmol/L. Next, MPTP was added to the cells separately. The CCK-8 method was used to measure the cell survival rate. Apart from the CCK-8 method, mitochondrial membrane potential detection, cell reactive oxygen species (ROS) detection, and other methods were also adopted to verify the effect of low, medium, and high concentrations of eleutheroside E on the MPTP-induced cell model. Western blot analysis was used to detect changes in the expression of intracellular proteins CytC, Nrf2, and NQO1 to clarify the mechanism. The results are as follows. Compared with the MPTP group, the survival rates of cells at low, medium, and high concentrations of eleutheroside E all increased. The mitochondrial membrane potential at medium and high concentrations of eleutheroside E increased. The ROS levels at medium and high concentrations of eleutheroside E decreased. Moreover, the apoptosis rate decreased and the expression levels of the intracellular proteins CytC, Nrf2, and NQO1 were upregulated. Conclusion: Eleutheroside E can improve the MPTP-induced apoptosis of PC-12 cells by increasing the mitochondrial membrane potential and reducing the level of intracellular reactive oxygen species (ROS). Moreover, the apoptosis of cells is regulated by the expression of CytC, Nrf2, and NQO1 proteins.

## 1. Introduction

Parkinson’s disease (PD) is a common progressive and degenerative disorder that affects the central nervous system. It is caused by a combination of age, genetics, inflammatory and oxidative stress, and environmental factors. Symptoms associated with Parkinson’s disease include both motor and non-motor symptoms. Parkinson’s disease is marked by the death of dopamine-producing neurons in the brain, especially in the substantia nigra and striatum. Its pathological feature is that the substantia nigra and striatum of the brain show pathological changes to the protein plaque of α-synuclein [1,2]. In the past decade, biological sex, age, heredity, environment, aging, and immunity have become important factors in the pathogenesis of PD. The main causes include proteasome dysfunction, mitochondrial dysfunction, oxidative stress, inflammatory reaction, etc. In clinical studies, it was found that the major clinical manifestation in PD patients was dyskinesia, which mainly had three physical signs: tremor, rigidity, and bradykinesia. In the early stage of PD, patients would experience some non-motor symptoms, including daytime sleepiness, urinary dysfunction, constipation, erectile dysfunction, and depression. However, in the late stage of PD, the motor and non-motor symptoms are obvious and difficult to treat; they include axial movement symptoms, hyposmia, depression, gait freezing, dysphagia, and speech disorders. PD is difficult to treat because the current diagnosis and treatment methods of PD are insufficient and the diagnostic indicators are uncertain. Moreover, delivery of the therapeutic drug molecules to the brain is frequently obstructed by the blood-brain barrier. Patients may experience drug resistance or side effects from the treatment drugs. The latest research findings suggest that PD may originate in the intestine. The abnormal synaptic nucleoprotein of PD has been shown to appear in the intestinal neurons before it appears in the brain. This indicates that the role of intestinal neurons is as important as that of the dopaminergic neurons [3]. This provides new ways of PD diagnosis and treatment, helps identify the changing process of the disease, and brings new opportunities for PD patients [4]. As the side effects from the current medication used to treat PD are severe, new alternatives should be found to reduce them.

Traditional Chinese medicine has been widely used for centuries to treat PD. *Acanthopanax senticosus* is widely used in China, South Korea, Japan, Russia, and other countries because of its high medicinal value for anti-fatigue, anti-inflammatory, anti-ulcer, anti-stress, and cardiovascular protection [5]. In the past two decades, scholars around the world have focused on *Acanthopanax senticosus* from the perspective of different disciplines, such as chemistry, pharmacology, and clinical medicine. Its reported compounds include triterpenoid saponins, lignans, coumarins, and flavonoids, among which the phenolic compounds are considered to be the most active compounds. Its phenolic compounds include syringin and *Acanthopanax senticosus* glycosides B and E. A large number of in vitro and in vivo experiments have proved that *Acanthopanax senticosus* can offer anti-stress, anti-inflammatory, anti-cancer, anti-ulcer, and liver protection [6,7]. The pharmacological action of *Acanthopanax senticosus* is similar to that of ginseng and it is almost non-toxic. Patients will not have adverse reactions, even after long-term treatment. Its role in PD treatment may involve regulating tyrosine metabolism, the oxidation of long-chain saturated fatty acid mitochondria, fatty acid metabolism, methionine metabolism, and sphingolipids metabolism [8,9,10]. Eleutheroside E (EE) is extracted from the dried rhizome of *Acanthopanax senticosus*, the saponins extracted together with EE also include the saponins A, B, C, D, F, and G. *Acanthopanax senticosus* saponins have various effects, such as dilating blood vessels, improving blood circulation, reducing tissue oxygen consumption, the two-way regulation of blood pressure, clearing oxygen free radicals, increasing the content of superoxide dismutase, etc. [5]. The extract of *Acanthopanax senticosus* contains the active ingredient eleutheroside E, which significantly enhances the behavior of the PD mouse model [5]. The researchers also incubated *Acanthopanax senticosus* extract with the MAO-B protein via affinity chromatography and mass spectrometry. They selected several monomeric compounds with high binding strength. The compounds included isoaziridine, chlorogenic acid, *Acanthopanax senticosus* E, and oleanol glucoside. Among them, *Acanthopanax senticosus* E has the highest binding strength with MAO-B. This indicates that *Acanthopanax senticosus* E has a good inhibitory effect on MAO-B [11]. Since EE has been shown to inhibit monoamine oxidase B (MAO-B) and reduce inflammation and oxidation, it has the potential to be a drug for alleviating and treating PD [12]. However, it is still unclear how EE protects the dopaminergic neurons. Although MPTP (1-methyl-4-phenyl-1,2,3,6-tetrahydropyridine) is a well-recognized neurotoxin [13], the compound itself is not toxic. When MPTP is ionized by monoamine oxidase B, the dopaminergic neurons can be damaged, creating a PD model [14]. This experiment explored the effect and mechanism of EE on an MPTP-induced PD cell model, in which EE was used as an intervention drug. This study aims to provide experimental data about the effect of EE on the PD cell model to support further research.

## 2. Results

### 2.1. Tolerance Limit of Cells to EE and Cell Modeling

**Figure 1:**
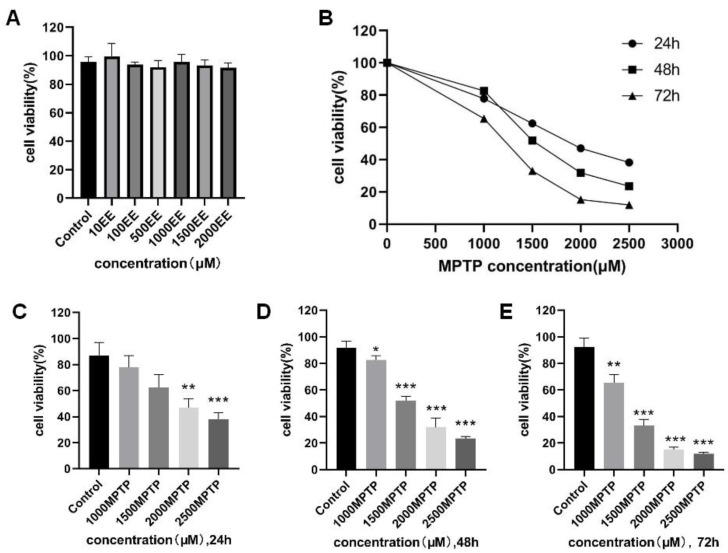
Test results of the cell tolerance limit to EE (**A**); the effect of different concentrations of MPTP and the time of action on the survival rate of PC-12 cells, the broken line diagram of MPTP for 24 h, 48 h, 72 h (**B**); MPTP action after 24 h (**C**); MPTP action after 48 h (**D**); MPTP after 72 h (**E**). Note: * *p* < 0.05, ** *p* < 0.01, and *** *p* < 0.001, compared with the control group.

**Figure 2:**
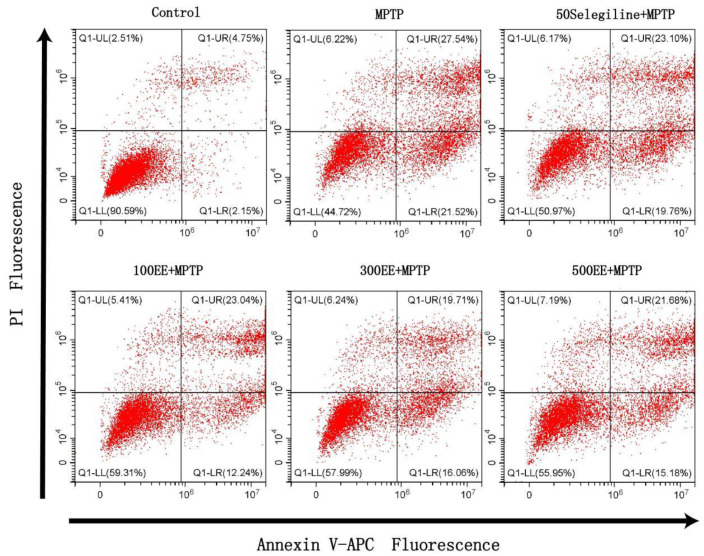
The results of the apoptosis rate of the PD oxidative stress cell model, as detected by flow cytometry, showed that Q1-LL had a normal cell population, Q1-UL had a necrotic or late apoptotic cell population, Q1-LR had an early apoptotic cell population, and Q1-UR had a late apoptotic cell population.

The tolerance of the cells was confirmed by the concentrations of EE in the seven groups; 10–2000 μM concentrations of EE are within the tolerance range of the cells. Within the range, no significant decrease was observed in cell viability (Figure 1). In addition, the MPTP-induced PD cell model also determined the induction conditions through a cell viability test. For the cell viability to be below 40%, the induction conditions included: 24 h, 2500 μM MPTP; 48 h, 2000 μM MPTP; 48 h, 2500 μM MPTP; 72 h, 1500 μM MPTP; 72 h, 2000 μM MPTP and 72 h, 2500 μM MPTP. After considering the time period and cost of the procedure, this experiment adopted 24 h, 2500 μM MPTP as the induction condition. The experiment cycle was set at 4 days to better control the state of the cells. Figure 2 shows the findings.

### 2.2. Effect of EE on the Survival Rate of PD Model Cells

The CCK8 method was adopted to reflect the cell viability. According to the results, in the MPTP group, cell viability was significantly lower (*p* < 0.001) and the apoptosis rate was significantly higher (*p* < 0.001) than in the control group. When compared to the MPTP group, the cell viability in the positive group and the EE low-, medium-, and high-concentration groups was significantly higher (*p* < 0.001), while the apoptosis rate was significantly lower (*p* < 0.05). The flow cytometer detection of APC and PI double-staining also showed similar results. Table 1 and Figure 2 show the findings.

### 2.3. Effects of EE on Mitochondrial Membrane Potential and Reactive Oxygen Species in PD Models

**Figure 3:**
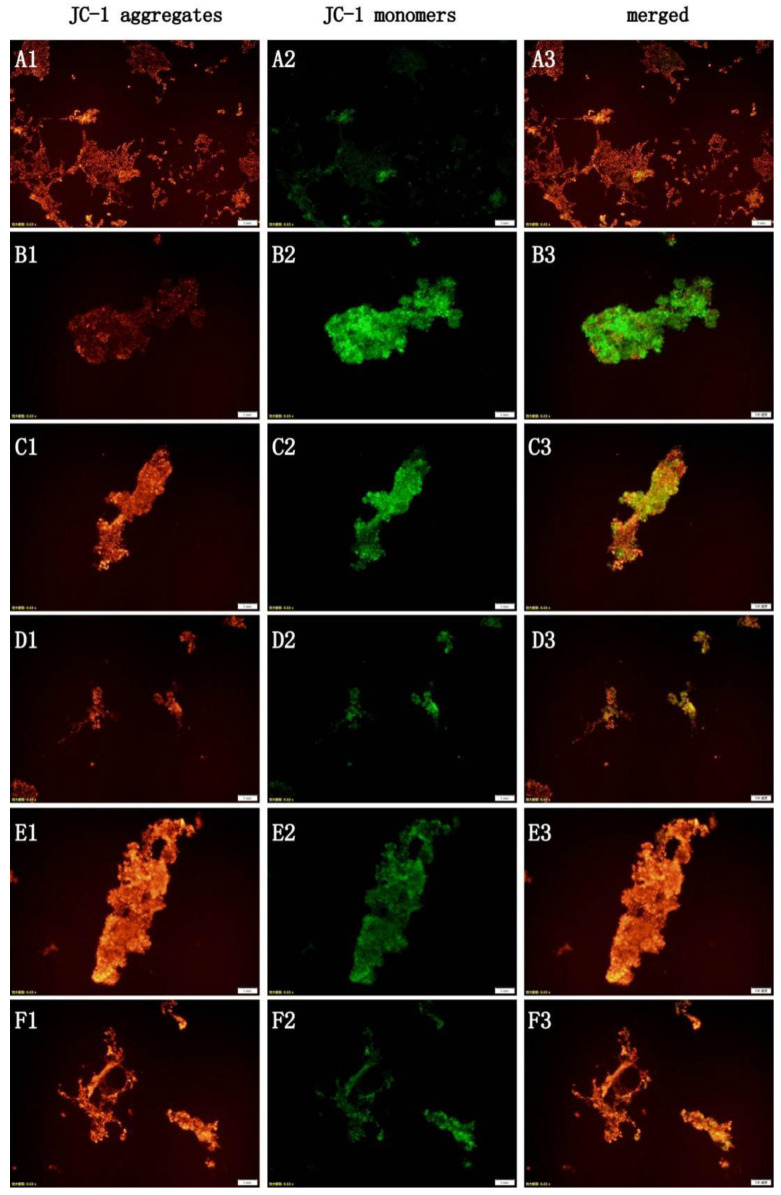
Mitochondria membrane potential of different groups with the JC-1 kit (**A**: control group; **B**: MPTP group; **C**: positive group; **D**: EE low-concentration group; **E**: EE medium-concentration group; **F**: EE high-concentration group; 1: JC-1 aggregates (red); 2: JC-1 monomers (green); 3: mitochondria after the cells received the standard merge treatment. Scale = 1 mm).

The mitochondrial membrane potential was detected using the JC-1 method; an ROS kit was used to detect the intracellular reactive oxygen species. When compared with the control group, the mitochondrial membrane potential decreased by 88% (*p* < 0.001) and the intracellular active oxygen level increased by 154% (*p* < 0.001) in the MPTP group. When compared with the control group, the mitochondrial membrane potential in the EE medium- and high-concentration groups significantly decreased by 41% and 53% (*p* < 0.001). The decrease was less than that in the MPTP group. When compared with the MPTP group, the EE medium- and high-concentration groups’ intracellular ROS content significantly decreased by about 22% and 19% (*p* < 0.01). There was no significant change in mitochondrial membrane potential and intracellular ROS levels in the positive group and the EE low-concentration group. The results can be seen in Table 2 and Figure 3.

### 2.4. Effects of EE on the Protein Expressions of CytC, Nrf2, and NQO1 in the PD Model

**Figure 4:**
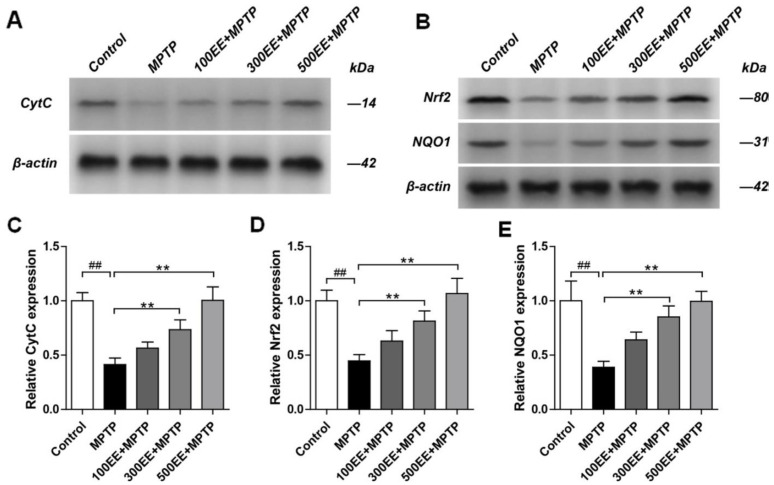
Representative Western blots are shown for the expression of CytC and the quantitation of the ratio of CytC in the cytoplasm (**A**,**C**); representative Western blots are shown for the general expressions of Nrf2 and NQO1 (**B**); quantitation of the ratio of Nrf2 and NQO1 (**D**,**E**). (X ± S; *n* = 3); ** *p* < 0.01 vs. the MPTP group; ## *p* < 0.01 vs. the control group.

The Western blot method was used to detect the expression of CytC, Nrf2, and NQO1 proteins in the cells. Compared with the control group, the protein expression levels of CytC, Nrf2, and NQO1 in the MPTP group were significantly downregulated (*p* < 0.01). Compared with the MPTP group, the protein expression levels of CytC, Nrf2, and NQO1 in the EE medium- and high-concentration groups significantly increased (*p* < 0.01). The outcomes are shown in Figure 4.

## 3. Materials

### 3.1. Cells

Rat adrenal pheochromocytoma cells (PC-12) were purchased from the cell bank of the Shanghai Institute for Biological Sciences (accession number: TCR 8). This cell line is constructed in China.

### 3.2. Main Reagents and Instruments

**Figure 5:**
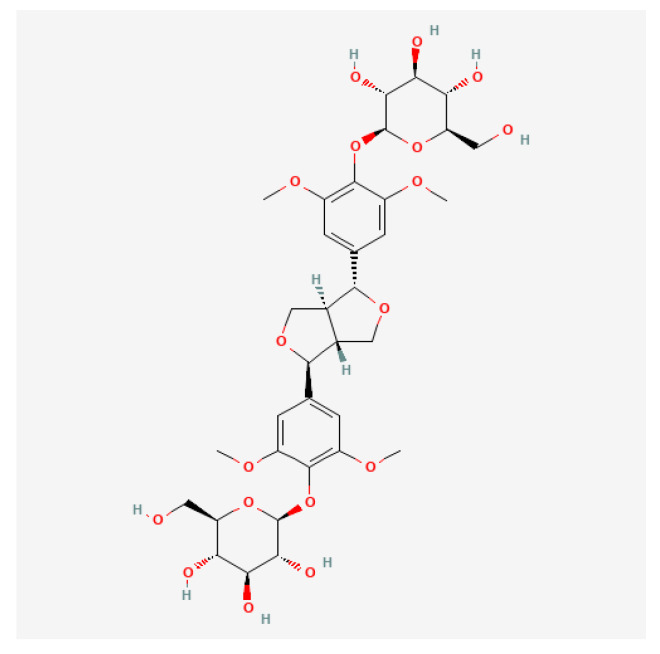
The eleutheroside E structure, with the molecular formula C34H46O18 and a molecular weight of 742.72.

The main reagents used were: eleutheroside E (as shown in Figure 5) (batch no.: P14D9F77612, Shanghai Yuanye Bio-Technology Co., Ltd., Shanghai, China), DMEM culture medium, fetal bovine serum (Gibco Co., Ltd., New York, NY, USA), penicillin, and streptomycin (Shanghai Yuanye Bio-Technology Co., Ltd., Shanghai, China). The testing kits used were: CCK-8 testing kit (Beyotime Biotechnology, Shanghai, China), mitochondrial membrane potential (ΔΨm) testing kit (batch no.: 20201014: Beijing Solarbio Life Sciences Co., Ltd., Beijing, China), cellular reactive oxide species (ROS) detection kit (batch no.: 20201215, Nanjing Jiancheng Bioengineering Institute, Nanjing, China), Annexin V-FITC/PI apoptosis detection kit (batch no.: 20201023, Beijing Solarbio Life Sciences Co., Ltd., Beijing, China).

The instruments used were an inverted fluorescence microscope (Olympus IX73, Tokyo, Japan) and a CytoFLEX flow cytometer (Beckman Coulter, Inc., Carlsbad, CA, USA).

## 4. Methods

### 4.1. Determination of Tolerance Limit of Cells to EE and Cell Modeling

Cells’ tolerance limits for polysaccharide compounds should be noted because high concentrations of polysaccharide compounds will affect the cells’ survival rates. Therefore, it is necessary to perform experiments to confirm the tolerance limit of PC-12 cells to EE. This is to ensure that when EE is added for intervention experiments, it will not produce toxic effects on PC-12 cells. To determine the tolerance range of PC-12 cells to EE, this paper used 6 concentrations of EE to test the cytotoxicity of EE. The experiment was divided into 7 groups: the control group, the 10EE group, the 100EE group, the 500EE group, the 1000EE group, the 1500EE group, and the 2000EE group.

As it is important to establish a reliable PD cell model, this experiment can only be carried out in appropriate induction conditions. To determine the appropriate induction dose of MPTP, three groups of MPTP concentrations were adopted: 1500 μM, 2000 μM, and 2500 μM. Three time durations (24 h, 48 h, and 72 h) were used to determine the appropriate induction time. After comparing the experimental time, this paper confirms the cell survival rate, which is less than 40%, and the appropriate dose of MPTP. Finally, a reasonable PD cell model induction condition was set up.

### 4.2. Preparation and Concentration Screening of EE

To prepare EE working liquors of different concentrations, it is necessary to prepare the EE mother solution in advance. The mother solution, with a concentration of 1000 μmol/L, was prepared from 3.71 mg of EE in 5 mL of DMEM complete medium containing 10% fetal bovine serum. The mother solution was stored at 4 °C in the dark and was changed once every two weeks. Before each use, the mother solution was diluted with DMEM complete medium, which contains 10% fetal bovine serum, to the required concentration. Solutions with EE concentrations of 100 μM, 300 μM, and 500 μM (μmol/L) were selected as the optimal concentration ranges for the treatment of PD model cells.

### 4.3. Cell Culture and Grouping

The PC-12 cells were cultured in DMEM with 10% fetal bovine serum and 1% cyan-streptomycin, which was changed every 2 days. Trypsin was added for digestion and passage when the cells were approximately 80% confluent.

The EE dosing experimental groups were divided into seven groups: the blank group, the control group, the positive group, the MPTP group, and groups with low (100 μM), medium (300 μM), and high (500 μM) EE doses.

### 4.4. Cell Viability

The CCK8 method was adopted to detect cell viability. PC-12 cells were taken and prepared as 8 × 10^5^ cells/mL single-cell suspension, which was inoculated into 96-well plates. Each 100 μL measure per well was cultured until the cells adhered to the wall. Then, the corresponding drug interventions and modeling treatments were performed for each group. Before the test, 10 μL of CCK-8 reagent was added and incubated for 3 h. Next, the absorbance values of each well at 450 nm were detected using a microplate reader.

Cell viability (%) = (OD drug group − OD blank group)/(OD control group − OD blank group) × 100%

### 4.5. Detection of Apoptosis Rate by Flow Cytometry

Flow cytometry was used to detect the viability of APC and PI double-stained cells. The PC-12 cells (5 × 10^5^ cells/mL) were inoculated into 6-well plates (1 mL per well) and cultured for 24 h until the cells adhered to the wall. The old culture medium was discarded and the corresponding drug intervention and modeling treatment were performed for each group. The old culture medium was discarded and the cells were collected after being washed with DPBS. First, 5 μL of Annexin V-APC dye was added, then it was followed by 5 μL of PI dye. Finally, APC and PI double fluorescence channels were selected in the flow cytometry for detection.

### 4.6. Detection of Mitochondrial Membrane Potential

The JC-1 method was adopted to detect the mitochondrial membrane potential. The PC-12 cells (5 × 10^5^ cells/mL) were inoculated into 6-well plates (1 mL per well) and cultured for 24 h until the cells adhered to the wall, then the old culture medium was discarded. The corresponding drug intervention and modeling treatment were then performed for each group and the old culture medium was discarded. DPBS was used for washing and the working solution for JC-1 staining was incubated at 37 °C for 20 min before the supernatant was discarded. After washing with the JC-1 staining buffer, 1 mL of culture solution was added. Then, the experimental results were observed, and photographs were taken under a fluorescence microscope. ImageJ software was used to measure the average fluorescence intensity.

### 4.7. Detection of Cellular Reactive Oxygen Species (ROS)

Detection of intracellular reactive oxygen species was performed using the ROS kit. The PC-12 cells (5 × 10^5^ cells/mL) were inoculated into 6-well plates (1 mL per well) and cultured for 24 h until the cells adhered to the wall. Then, the old culture medium was discarded. The corresponding drug interventions and modeling treatments were performed for each group and the old culture medium was discarded. A working solution containing DCFH-DA was added and incubated in a cell incubator at 37 °C for 1 h. The cells were collected after being washed with DPBS. The fluorescence channel of FITC was selected for detection by flow cytometry.

### 4.8. Detection of Protein Expressions of CytC, Nrf2, and NQO1 by Western Blot Analysis

The Western blot analysis method is used to detect the expression of the CytC, Nrf2, and NQO1 proteins in cells. PC-12 cells (5 × 10^5^ cells/mL) were inoculated into 6-well plates (1 mL per well) and cultured for 24 h until the cells adhered to the wall. Then, the old culture medium was discarded, and the corresponding drug interventions and modeling treatments were performed for each group. Next, the old culture medium was discarded, and the cells were collected after being washed with DPBS. After 1 mL of lysed RIPA cells supplemented with PMSF were added, the cells were centrifuged at 12,000 *g* for 5 min at 4 °C and the supernatant was collected for protein quantification using the BCA method. Then, 50 μg of total protein was added. After gel electrophoresis was complete, the result was transferred to a PVDF membrane. The membrane was stained with Ponceau S dye solution and cleaned by TBST for further observation of the proteins. Anti-CytC (1:1000), Anti-Nrf2 (1:1000), Anti-NQO1 (1:1000), and GAPDH (1:1000) antibodies were diluted to appropriate concentrations. After TBST was added, they were incubated overnight at 4 °C. The membrane was washed with TBST three times (for 10 min each time) and added to goat anti-rabbit IgG at the appropriate concentration. Then, it was incubated for 1 h at room temperature. The ECL hypersensitive chemiluminescence kit was used for development.

## 5. Discussion

Traditional Chinese medicines (TCMs) and their impact on neurodegenerative illnesses have been the subject of numerous investigations for many years. Traditional Chinese medicine monomers can treat neurodegenerative diseases and effectively alleviate the complications and side effects caused by Western medicines [15,16,17]. Patients who are resistant to Western medicines or who are troubled by the medicines’ side effects can resort to TCMs for treatment and the alleviation of symptoms. Many TCMs and their active ingredients, such as curcumin, gastrodin, baicalein, ginkgolide, and ginsenoside, offer good neuroprotective effects [18,19,20]. This study aims to provide an experimental basis for clinical practice and to demonstrate the need for further research into the neuroprotective effects of EE and other new, effective monomers.

According to the results of the current experiment, PC-12 cells showed good tolerance of EE. In other words, the cells’ survival rate would not decrease with an effective dose of EE, as long as the dose of EE was within the appropriate range. In addition, MPTP was used to determine the appropriate PD cell model conditions through a cell model screening experiment. The results will provide supporting data for a subsequent study of the PD cell model. After being exposed to MPTP, the PC-12 cells had a significantly lower survival rate and a significantly higher apoptosis rate, indicating that a cell model of PD was successfully established. As seen in the results of CCK8 detection and flow cytometry, after pretreatment with medium and high concentrations of EE, the cell survival rates of the two groups significantly increased and the apoptosis rate significantly decreased. The results indicate that EE had a certain protective effect on the PD cell model.

Recent research has shown that disturbance of mitochondrial function will lead to cell apoptosis, as various macromolecules in the mitochondria will affect or even reduce the membrane potential [21,22]. Moreover, higher levels of intracellular ROS also lead to the destruction of cell mitochondria and apoptosis [23,24,25,26,27]. The results of this experiment showed that the medium and high concentration of EE could effectively improve the mitochondrial membrane potential of PD model cells and reduce the intracellular active oxygen level. Western blot detection of the protein expression levels showed that EE upregulated the expression of CytC, Nrf2, and NQO1 in a concentration-dependent manner in the PD cell model. The results indicated that an increase in CytC, Nrf2, and NQO1 proteins could effectively suppress apoptosis in the PD cell model. However, after the therapeutic effect reached a maximum level, further increasing the drug concentration could not generate additional benefits. In this study, the effect of EE on the PD cell model was tested via CCK-8 cell viability measurement. The PD cell model showed that the cell viability of the medium-concentration EE group was higher than that of the high-concentration EE group. In the detection of mitochondrial membrane potential, the reduction in mitochondrial membrane potential in the medium-concentration EE group was less than that in the high-concentration EE group. As for the detection of intracellular reactive oxygen species, the PD cell model showed that the increase in intracellular reactive oxygen species in the medium-concentration EE group was less than that in the high-concentration EE group, while the results of flow cytometry showed that the cell apoptosis rate in the medium-concentration EE group was less than that in the high-concentration EE group. Therefore, the effect of the medium-concentration EE group on the PD cell model was the most significant. Furthermore, this indicated that EE had a therapeutic window in the PD cell model [28]. A similar situation was also found when studying the effects of other polysaccharide monomer compounds on PD cell models [29]. Further research is needed to confirm the effect window that is produced by EE on PD cell models. This is also one of the problems yet to be solved in this research at this stage.

## 6. Conclusions

In summary, this study finds that Eleutheroside E can improve the MPTP-induced apoptosis of PC-12 cells by increasing the mitochondrial membrane potential and reducing intracellular ROS levels. In addition, in the EE medium- and high-concentration groups, the protein expressions of CytC, Nrf2, and NQO1 in the PD cell model changed during cell apoptosis after EE administration. This is only part of the protein expression of the protective effect of Eleutheroside E on PD cells; therefore, more studies are required to confirm the results, while the finding regarding protein expression changes can serve as a reference. Moreover, this study provides an experimental basis for the establishment of a PD cell model and the application of EE in the PD cell model. It also provides a reference for the use of MPTP when establishing the PD model cells. Moreover, the necessity of the discovery and utilization of monomers and the active ingredients of traditional Chinese medicine was further explained. However, this result can only prove that EE has a protective effect on PD cell models. For better application, further studies are needed to confirm that EE has the same effect in vivo. Although EE is safe in PD cell models, further studies are needed to prove that EE is safe for use by humans. In addition, EE, as with the current mainstream PD drugs, can only play a weak protective role in PD cell models. In other words, it can only slow down rather than prevent the deterioration and death seen in PD cell models. Therefore, the authors hope that more effective drugs can be found as a cure for Parkinson’s disease.

## Figures and Tables

**Table 1:** PD cell model survival rates and apoptosis rates in different groups (mean ± SEM; *n* = 3).

Groups	Concentrations (μM)	Survival Rates (%)	Apoptosis Rates (%)
Control group	NA	100.00 ± 0.00	4.75 ± 0.83
MPTP group	NA	42.30 ± 1.98 ###	27.54 ± 2.51 ###
Positive group	50	49.46 ± 4.81 **	23.10 ± 1.36
EE low-concentration group	100	48.89 ± 1.80 **	23.04 ± 3.32
EE medium-concentration group	300	59.20 ± 3.13 ***	19.71 ± 1.77 **
EE high-concentration group	500	56.86 ± 2.31 ***	21.68 ± 2.04 *
F	NA	30.34	41.22
*p*	NA	0.001	0.001

Note: ### *p* < 0.001 vs. the control group, * *p* < 0.05, ** *p* < 0.01, and *** *p* < 0.001 vs. the MPTP group. NA: Not Available.

**Table 2:** Results of the mitochondria membrane potential and the ROS of different groups (mean ± SEM; *n* = 3).

Groups	Concentrations (μM)	JC-1 (ΔΨm) RFU	ROS
Control group	NA	10.42 ± 0.10	14,007.97 ± 252.44
MPTP group	NA	1.24 ± 0.243 ###	21,618.67 ± 324.15 ###
Positive group	50	3.51 ± 0.39 **	19,366.93 ± 597.30
EE low-concentration group	100	3.44 ± 0.32 **	19,786.57 ± 512.10
EE medium-concentration group	300	5.53 ± 0.27 ***	16,789.77 ± 158.48 **
EE high-concentration group	500	4.28 ± 0.37 ***	17,592.50 ± 342.17 **
F	NA	326.8	118.6
*p*	NA	0.001	0.003

Note: ### *p* < 0.001 vs. the control group; ** *p* < 0.01 and *** *p* < 0.001 vs. the MPTP group. NA: Not Available.

## Data Availability

The data are available from the corresponding author on reasonable request.

## Sample Availability

Samples of the compounds are not available from the authors.
